# Anti-type 2 monoclonal antibody therapy for eosinophilic COPD beyond triple therapy: a systematic review and meta-analysis with strict eosinophil inclusion criteria

**DOI:** 10.3389/fmed.2026.1895960

**Published:** 2026-07-22

**Authors:** Jialing Chen, Caiming Liang, Zhen Wang

**Affiliations:** 1 The First Affiliated Hospital of Zhejiang Chinese Medical University (Zhejiang Provincial Hospital of Chinese Medicine), Hangzhou, China; 2 The First School of Clinical Medicine, Zhejiang Chinese Medical University, Hangzhou, China; 3 Cixi Hospital of Traditional Chinese Medicine, Ningbo, China

**Keywords:** biologic therapy, chronic obstructive pulmonary disease, eosinophil, meta-analysis, monoclonal antibodies

## Abstract

**Background:**

As a heterogeneous disease, chronic obstructive pulmonary disease (COPD) presents with eosinophil driven type 2 inflammation in some patients. Monoclonal antibodies targeting this pathway have shown promising therapeutic effects in related COPD clinical trials, but updated evidence on their efficacy and safety is still needed, specifically in strictly defined eosinophilic COPD patients.

**Methods:**

This study searched 8 databases from inception to March 17, 2026, for randomized controlled trials (RCTs) comparing type 2 mAbs vs. placebo in eosinophilic COPD, defined as a blood eosinophil count (BEC) ≥300 cells/μL. The primary outcome was the annual rate of moderate-to-severe exacerbations; secondary outcomes covered changes in pre-bronchodilator forced expiratory volume in 1 s (pre-BD FEV_1_), St. George's Respiratory Questionnaire (SGRQ) score, Evaluating Respiratory Symptoms in COPD (E-RS: COPD) score, and safety-related events. A random effects model in RevMan was used for analysis. Risk of bias was assessed with the RoB 2.0 tool, and the certainty of evidence with the GRADE approach.

**Results:**

6 RCTs (pooled into 5 research units, as METREX and METREO were combined) involving 3,171 participants were included. Type 2 mAbs reduced the annual rate of moderate-to-severe exacerbations vs. placebo (RR = 0.73, 95% CI 0.66 to 0.80), with consistent subgroup efficacy. Among secondary outcomes, pre-bronchodilator FEV_1_ improved by 61.97 mL (95% CI 5.09 to 118.85). SGRQ score decreased by −3.16 points (95% CI −4.54 to −1.79). E-RS: COPD score decreased by −0.67 points (95% CI −1.20 to −0.14). There was no statistically significant difference between the treatment group and placebo group in the incidence of adverse events. Evidence certainty was high to moderate, downgraded due to indirectness and inconsistency.

**Conclusions:**

In eosinophilic COPD patients, type 2 mAbs reduce moderate-to-severe exacerbations without additional safety signals. Improvements in lung function and quality of life were statistically significant but modest. The symptom benefit was also statistically significant, but should be interpreted with caution given the non-robust sensitivity analysis. This evidence supports the use of type 2 mAbs as an add on treatment for these patients. Future studies should establish a uniform clinical threshold and integrate multiple biomarkers for precision phenotyping.

**Systematic review registration:**

https://www.crd.york.ac.uk/PROSPERO/view/CRD420261334527, identifier: CRD420261334527.

## 1 Introduction

Chronic obstructive pulmonary disease (COPD) is an inflammatory airway disease characterized by persistent, progressive airflow limitation and chronic respiratory symptoms. It has high morbidity and mortality, placing a heavy economic and social burden on the world (1, 2). Currently, first-line pharmacological treatment for COPD mainly consists of inhaled agents, including long-acting β_2_-agonists (LABA), long-acting muscarinic antagonists (LAMA), and inhaled corticosteroids (ICS). However, issues such as poor adherence and adverse drug reactions remain (3, 4). COPD is highly heterogeneous, and some patients may still experience frequent exacerbations even after receiving maximal-intensity triple inhaled therapy (LABA/LAMA/ICS), highlighting the notable efficacy limitations of conventional triple therapy in specific populations (5).

Chronic inflammation underlies the entire pathophysiological process of COPD. Unlike the traditional view centered on neutrophils, recent studies have indicated that type 2 inflammation, characterized by elevated eosinophils, is also an important factor in the pathogenesis and exacerbation of COPD (6, 7). At the pathophysiological level, eosinophils play a central driving role in acute exacerbations by releasing major basic protein, eosinophil cationic protein, and various pro-inflammatory cytokines. These mediators directly damage the airway epithelium, induce mucus hypersecretion, and enhance airway hyperresponsiveness (7). Clinically, approximately 20%−40% of patients have a type 2 inflammatory phenotype, which is associated with worse prognosis, a higher risk of exacerbations, and differential response to ICS therapy (8–10). These findings strongly suggest that type 2 inflammation is an important and modifiable mechanism in COPD. Given the successful application of targeting the type 2 pathway in asthma, another typical inflammatory airway disease, various type 2 monoclonal antibodies (type 2 mAbs), including anti-IL-4Rα, anti-IL-5/IL-5, and anti-IL-33, are being explored for the treatment of COPD (11–15). Existing randomized controlled trials and *post-hoc* analyses generally support the clinical benefit of type 2 mAbs in treating COPD, with potentially greater efficacy in eosinophilic COPD. Among these, dupilumab has been approved as an add-on maintenance treatment for patients with uncontrolled COPD of the eosinophilic phenotype. Yet, differences in targets of action, definitions of eosinophilic COPD, and population characteristics have led to some heterogeneity across studies. In particular, the lack of uniform eosinophil thresholds means that high-quality evidence to guide individualized treatment remains lacking (16–19).

This systematic review and meta-analysis precisely focus on randomized controlled trials (RCTs) of treatment with type 2 mAbs for eosinophilic COPD, defined as a blood eosinophil count (BEC) ≥300 cells/μL, systematically synthesizing available evidence. It aims to clarify the overall effects on reducing exacerbations, improving lung function and quality of life, as well as to explore potential sources of heterogeneity in treatment responses, thereby providing evidence-based support for optimizing precision treatment in this COPD subgroup.

## 2 Methods

### 2.1 Systematic review registration

This systematic review and meta-analysis was conducted and reported following the Preferred Reporting Items for Systematic Reviews and Meta-Analyses (PRISMA) 2020 statement (20). The expanded PRISMA 2020 checklist and the PRISMA 2020 abstract checklist are all available in Supplementary material. The study protocol was preregistered in the International Prospective Register of Systematic Reviews (PROSPERO) database (registration number: CRD420261334527).

### 2.2 Search strategy

This systematic search covered PubMed, Embase, Cochrane Library, Web of Science, China National Knowledge Infrastructure (CNKI), Wanfang Data Knowledge Service Platform, Chongqing VIP Information Chinese Science and Technology Journal Database, and China Biology Medicine disc (CBM) from their inception to March 17, 2026. The search strategy was independently developed and refined by two reviewers. Boolean operators were used to combine controlled vocabulary terms (e.g., MeSH and Emtree) and free-text words covering core concepts such as “COPD,” “monoclonal antibodies,” and “RCT.” The detailed search strategy is provided in Supplementary material. We also searched http://ClinicalTrials.gov and the WHO International Clinical Trials Registry Platform (ICTRP) for unpublished or ongoing studies, and screened the reference lists of included studies and relevant systematic reviews to avoid missing potentially eligible literature.

### 2.3 Eligibility criteria

The inclusion criteria for this study were established according to the PICOS framework as follows:

(1) Participants: Adult patients who met the diagnostic criteria for COPD, with a BEC of ≥300 cells/μL either at screening or at any point in the previous year, as determined by standard laboratory assays at screening and/or baseline for eligibility confirmation;
(2) Intervention: Monoclonal antibodies targeting the type 2 inflammatory pathway, with no restrictions on dose or treatment duration;
(3) Comparison: Placebo;
(4) Outcome: The annual rate of moderate-to-severe exacerbations during the 52-week trial period served as the primary outcome, and at least one of the following secondary outcomes was required to be reported: change from baseline in pre-bronchodilator forced expiratory volume in 1 s (pre-BD FEV_1_), change from baseline in St. George's Respiratory Questionnaire (SGRQ) score, or change from baseline in Evaluating Respiratory Symptoms in COPD (E-RS: COPD) score;
(5) Study design: Randomized controlled trial design.

### 2.4 Study selection and data extraction

Two researchers (Jialing Chen and Caiming Liang) independently performed literature screening and data extraction. Initially, articles clearly not meeting the PICOS criteria were excluded by reviewing titles and abstracts. For the remaining articles, full texts were retrieved, after which a second round of screening was conducted based on the inclusion and exclusion criteria described above. Disagreements during the screening process were resolved through discussion or consultation with a third researcher, Zhen Wang. The screening results were recorded in a PRISMA flow diagram. For the finally included studies, a standardized form was used to extract data including basic study information, patient characteristics, intervention details, outcome measures, and relevant subgroup data. When data were missing, priority was given to retrieving them from Supplementary materials of the original articles or from clinical trial registration records. If the data remained unavailable, they were marked as “not reported (NR)” and excluded from the pooled analysis of the corresponding outcome.

### 2.5 Data analysis

RevMan software (V.5.4.1, The Cochrane Collaboration, 2020) was used to perform the meta-analysis. For the primary outcome (The annual rate of moderate-to-severe exacerbations), the log rate ratio and its standard error (SE) were extracted. For secondary outcomes (FEV_1_, SGRQ, and E-RS: COPD), the sample size, mean, and standard deviation (SD) were extracted. Both types of outcomes were pooled using the inverse-variance method, with the final results presented as rate ratio (RR) or mean difference (MD) along with their 95% confidence intervals (95% CIs). Considering the potential heterogeneity across studies in terms of drug type, inclusion criteria, and follow-up duration, a random-effects model (DerSimonian and Laird) was used for all pooled analyses. For the primary outcome, subgroups were predefined based on age, sex, smoking status, BEC, and the number of prior exacerbations, whose effect sizes were pooled and compared using interaction tests. Three sensitivity analyses were then performed to assess the robustness of the conclusions, namely the leave-one-out analysis, the analysis restricted to studies with screening period BEC ≥300 cells/μL, and the fixed effects model analysis. For publication bias, when the number of included studies was sufficient (e.g., ≥10), Egger's regression test was conducted and a funnel plot was generated (21, 22). All statistical tests were two-sided, and a *P* value < 0.05 was considered statistically significant.

### 2.6 Risk of bias assessment and certainty of evidence

Methodological quality was assessed using the Cochrane Risk of Bias 2.0 tool. Two reviewers independently judged each of the five domains (randomization process, deviations from the intended interventions, missing outcome data, measurement of the outcome, and selective of the reported result) as low risk, some concerns, or high risk (23). Discrepancies were resolved through discussion or intervention by Zhen Wang. The GRADE approach was used to rate the certainty of evidence for the primary outcome and key secondary outcomes (high, moderate, low, or very low), with downgrading considerations including risk of bias, inconsistency, indirectness, imprecision, and publication bias (24).

## 3 Results

### 3.1 Eligible studies and characteristics

**Figure 1:**
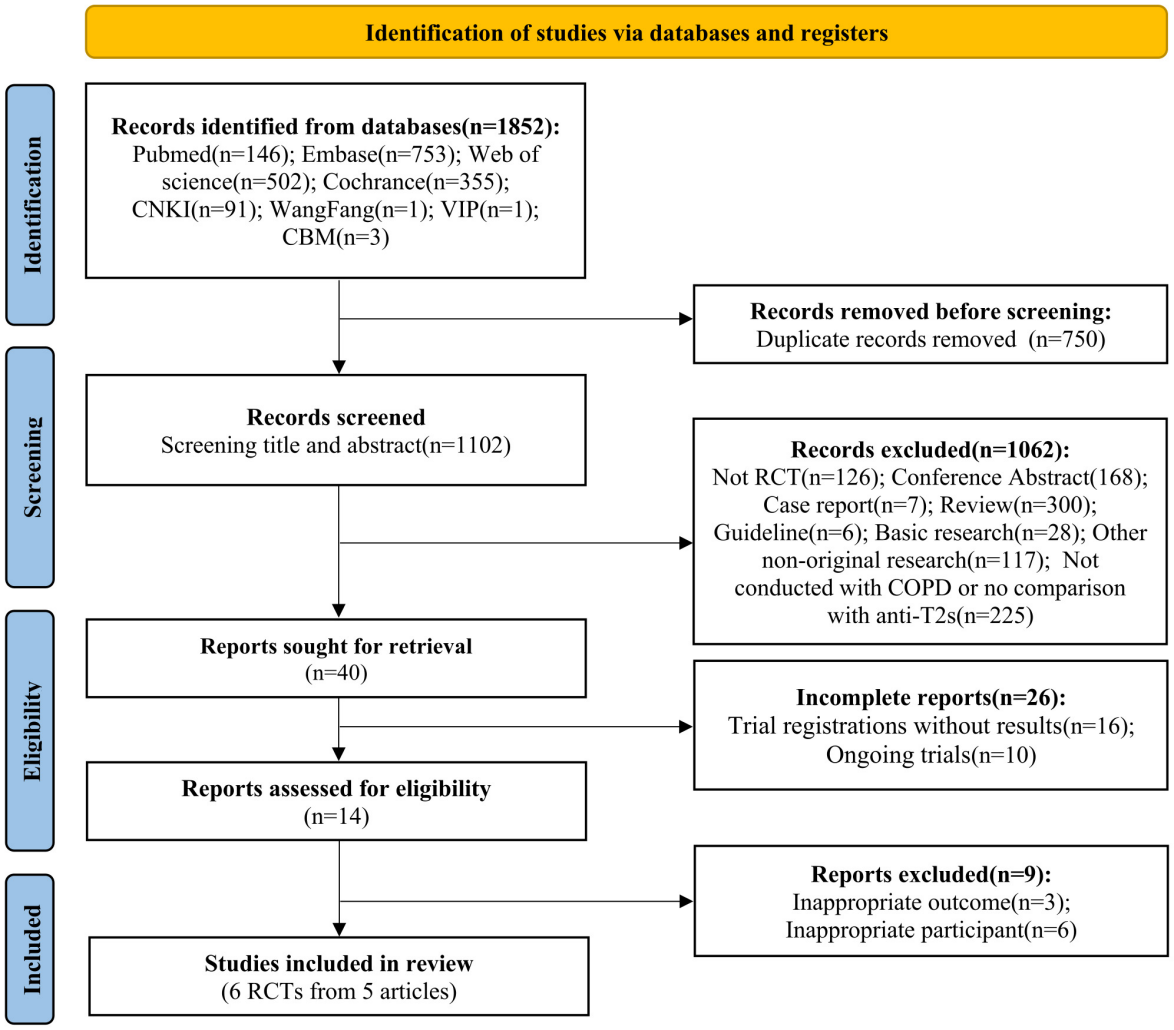
Systematic review and meta-analysis flow-diagram illustrating systematic search and screening strategy, including number of studies meeting eligibility criteria and number of excluded studies.

The initial search identified 1,852 records, of which 1,102 remained after duplicate removal. After screening titles, abstracts, and keywords, 1,062 records were excluded, leaving 40 records for full text review. Among these, 26 records were unavailable due to incomplete studies, while 14 records with full reports proceeded to eligibility assessment, and 9 articles were excluded due to mismatched outcomes or participant populations, as detailed in Supplementary material. Ultimately, 5 articles encompassing 6 RCTs were included in the meta-analysis (11, 13–15, 19). The complete screening process is presented in the PRISMA flow diagram (Figure 1).

The 6 included RCTs were published between 2017 and 2025, covering regions including Asia, Europe, and South America. BOREAS, NOTUS, and MATINEE directly enrolled patients with screening BEC ≥300 cells/μL, whereas COURSE, METREX, and METREO contributed subgroup data: COURSE using the screening BEC ≥300 subgroup, and METREX/METREO using the subgroup with BEC ≥300 at screening or in the prior year. And the primary reports of METREX and METREO provided pooled subgroup data for patients with BEC ≥300 cells/μL at screening or in the prior year on a 100 mg dose of mepolizumab. Based on this available subgroup data related to historical BEC, these two trials were treated as a single study unit, which contributed only to the primary exacerbation outcome and did not provide subgroup data for the secondary outcome. Therefore, this meta-analysis included 5 research units and 3,171 participants. Additionally, the MATINEE trial reported all results at 52-weeks and partial results at 104 weeks, and we extracted the 52-week data for analysis to maintain consistency with the follow up durations of the other included trials. The basic information and baseline characteristics of each trial are presented in Tables 1 and 2.

**Table 1:** General characteristics of included studies.

Study	Year	No. of countries	Trial number identifier	Participants, no.^a^	Drug	Dose and routine	Treatment duration, wks	Follow-up duration (post-treatment), wks	BEC entry criterion^b^
BOREAS (13)	2023	24	NCT03930732	T^c^: 468	Dupilumab	300 mg q2w, SC	52	12	①
				C^d^: 471					
COURSE (19)	2025	10	NCT04039113	T: 24	Tezepelumab	420 mg q4w, SC	48	12	①
				C: 32					
MATINEE (15)	2025	25	NCT04133909	T: 403	Mepolizumab	100 mg q4w, SC	52–104	4	①
				C: 401					
METREX + METREO (11)	2017	15	NCT02105961	T: 218	Mepolizumab	100 mg q4w, SC	52	8	②
			NCT02105948	C: 219					
NOTUS (14)	2024	29	NCT04456673	T: 470	Dupilumab	300 mg q2w, SC	52	12	①
				C: 465					

Q2w, every 2 weeks; Q4w, every 4 weeks; SC, subcutaneous injection.

^a^The number of participants included in the meta-analysis, rather than the total number of participants in the trial.

^b^Criteria for inclusion in the meta-analysis population; BEC ≥300 cells/μL at screening; BEC ≥300 cells/μL at screening or in the prior year.

^c^T = Treatment group.

^d^C = Control group.

**Table 2:** Baseline characteristics of study populations^a^.

Study	Male sex, no. (%)	Age, yr	BMI, kg/m^2^	Current smoker, no. (%)	BEC (cells/μl)^b^	Post-BD FEV_1_		SGRQ score
						Volume, liters	Percent of predicted value	
BOREAS (13)	T: 298 (63.7)	T: 65.0 ± 8.0	T: 27.5 ± 5.4	T: 134 (28.6)	T: 394 ± 261	T: 1.39 ± 0.47	T: 50.6 ± 13.3	T: 48.4 ± 17.0
	C: 322 (68.4)	C: 65.2 ± 8.1	C: 27.6 ± 5.7	C: 148 (31.4)	C: 408 ± 331	C: 1.41 ± 0.47	C: 50.6 ± 13.0	C: 48.4 ± 17.8
COURSE (19)	NR	NR	NR	NR	NR	NR	NR	NR
MATINEE (15)	T: 276 (68.5)	T: 66.4 ± 8.1	T: 27.4 ± 5.3	T: 111 (27.5)	T: 480 ± 0.378	T: 1.31 ± 0.48	T: 48.1 ± 15.7	T: 55.3 ± 17.7
	C: 275 (68.6)	C: 66.0 ± 7.9	C: 27.1 ± 5.5	C: 111 (27.7)	C: 480 ± 0.398	C: 1.34 ± 0.54	C: 48.2 ± 15.8	C: 53.9 ± 17.9
METREX+ METREO (11)	NR	NR	NR	NR	NR	NR	NR	NR
NOTUS (14)	T: 320 (68.1)	T: 65.2 ± 8.1	T: 28.1 ± 5.3	T: 142 (30.2)	T: 412 ± 357	T: 1.43 ± 0.49	T: 49.5 ± 12.6	T: 52.0 ± 17.5
	C: 312 (67.1)	C: 64.9 ± 8.5	C: 27.8 ± 5.6	C: 134 (28.8)	C: 402 ± 314	C: 1.46 ± 0.50	C: 50.7 ± 12.6	C: 51.1 ± 16.5

BMI, body mass index; BD, bronchodilator; BEC, blood eosinophil count; FEV_1_, forced expiratory volume in 1 s; SGRQ, St. George's respiratory questionnaire; NR, not reported.

^a^Plus–minus values indicate mean ± SD, unless otherwise indicated.

^b^BOREAS and NOTUS reported arithmetic mean ± SD; MATINEE, METREX, and METREO reported geometric mean ± logSD.

### 3.2 Risk of bias assessment

**Figure 2:**
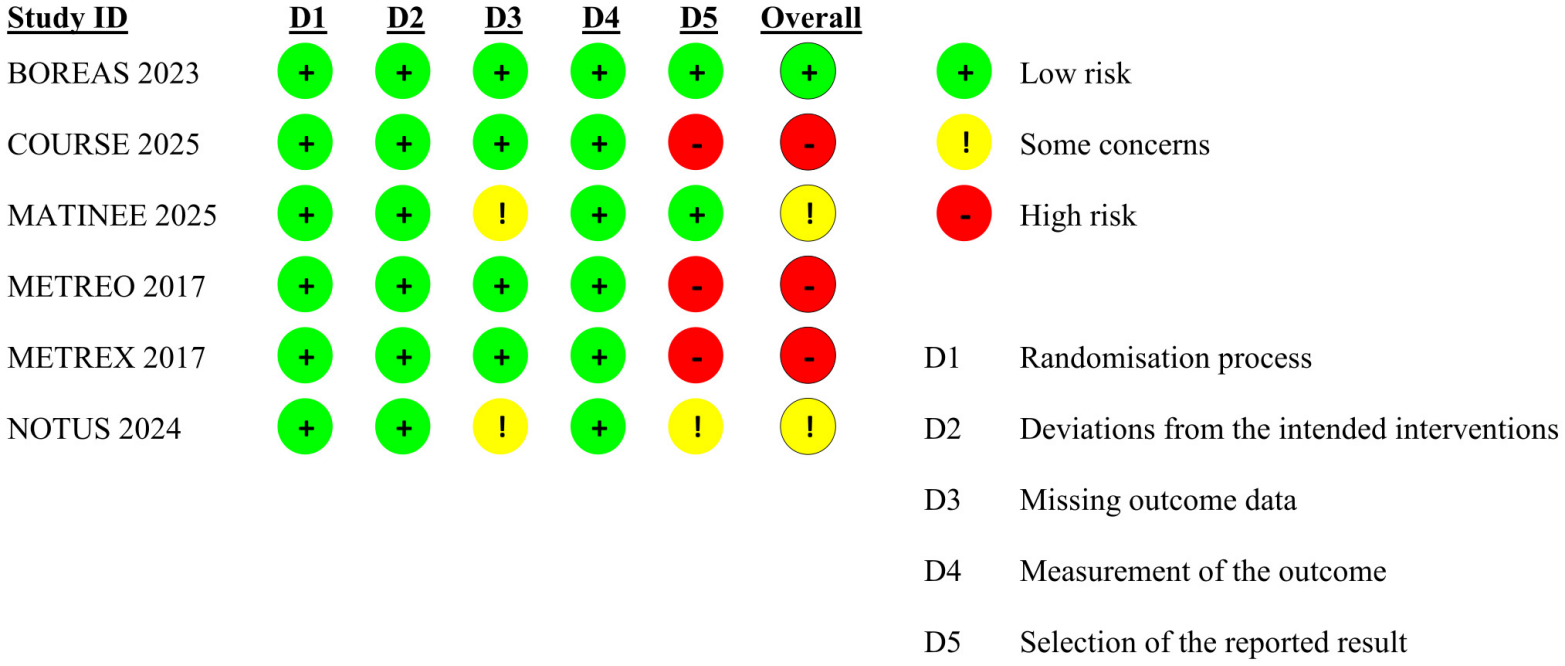
Risk of bias chart.

The methodological quality of the included RCTs varied, as shown in Figure 2. BOREAS was rated as low risk, whereas COURSE, METREO, and METREX were rated as high risk overall due to reporting bias. For MATINEE and NOTUS, despite the use of multiple imputation, considerable missing outcome data led to a judgment of some concerns in the domain of missing outcome data. Additionally, NOTUS prominently reported subgroup findings in *post hoc* analyses, raising the possibility of selective reporting after the fact. As a result, both trials received an overall rating of some concerns.

### 3.3 Meta-analysis results

#### 3.3.1 Annualized rate of moderate-to-severe exacerbations

##### 3.3.1.1 Overall results

**Figure 3:**
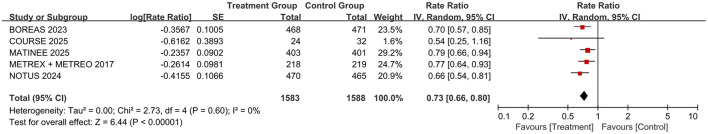
Forest plot of the annualized rate of moderate-to-severe exacerbations.

5 research units reported the efficacy of type 2 mAbs in reducing the annual rate of moderate-to-severe exacerbations in COPD (Figure 3). A random-effects meta-analysis showed that, compared with placebo, type 2 mAbs reduced the annual rate of moderate-to-severe exacerbations (*k* = 5, *n* = 3,171; RR = 0.73, 95% CI 0.66 to 0.80; *P* < 0.00001). Heterogeneity tests indicated no substantial heterogeneity across studies (*I*^2^ = 0%, τ^2^ = 0.00, *P* = 0.60). The point estimates of all included studies fell to the left of the line of no effect (RR < 1), uniformly supporting a treatment benefit.

##### 3.3.1.2 Subgroup analysis

**Figure 4:**
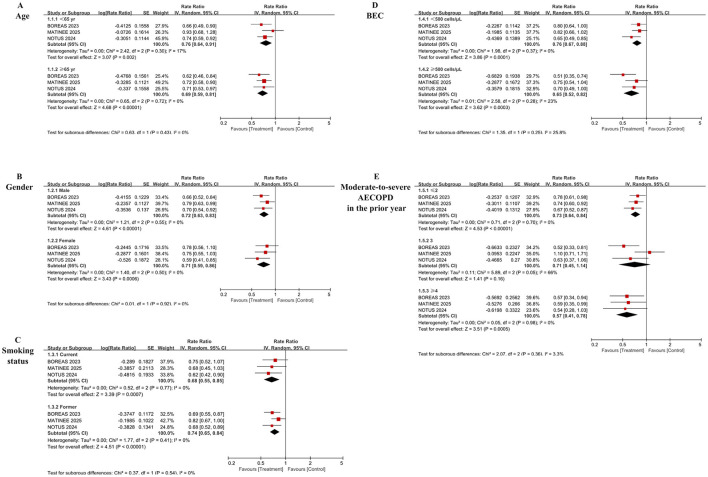
Forest plot of the subgroups with annualized moderate-to-severe exacerbation rate. **(A)** Age, **(B)** Gender, **(C)** Smoking status, **(D)** BEC, **(E)** Moderate-to-severe AECOPD in the prior year.

To assess the consistency of efficacy across different populations, subgroup analyses were conducted based on age, sex, smoking status, BEC, and the number of moderate-to-severe exacerbations in the year prior to enrollment (Figure 4). For age, the

RR was 0.76 (95% CI 0.64 to 0.91, *P* = 0.002) for patients aged < 65 years and 0.69 (95% CI 0.59 to 0.81, *P* < 0.00001) for those aged ≥65 years (*P* for interaction = 0.43). For sex, the RR was 0.72 (95% CI 0.63 to 0.83, *P* < 0.00001) for males and 0.71 (95% CI 0.59 to 0.86, *P* = 0.0006) for females (*P* for interaction = 0.92). Regarding smoking status, current smokers had a RR of 0.68 (95% CI 0.55 to 0.85, *P* = 0.0007) and former smokers had a RR of 0.74 (95% CI 0.65 to 0.84, *P* < 0.00001) (*P* for interaction = 0.54). Based on baseline BEC, the RR was 0.76 (95% CI 0.67 to 0.88, *P* = 0.0001) for patients with BEC < 500 cells/μL and 0.65 (95% CI 0.52 to 0.82, *P* = 0.0003) for those with BEC ≥500 cells/μL (*P* for interaction = 0.25). For the frequency of moderate-to-severe exacerbations in the prior year, the RR was 0.73 (95% CI 0.64 to 0.84, *P* < 0.00001) for ≤ 2 exacerbations, 0.71 (95% CI 0.45 to 1.14, *P* = 0.16) for 3 exacerbations, and 0.57 (95% CI 0.41 to 0.78, *P* = 0.0005) for ≥4 exacerbations, with a *P* for interaction of 0.36.

Overall, none of the above results showed significant subgroup interactions, indicating great concordance of the treatment effect. Of note, the subgroup with 3 exacerbations per year exhibited moderate heterogeneity (*I*^2^ = 66%) and the pooled result did not reach statistical significance. This may be attributable to the fact that the effect direction in the MATINEE trial (RR = 1.10) in this subgroup was opposite to that of the others. Nevertheless, the interaction test still indicated no statistically detectable difference.

#### 3.3.2 Lung function: change in pre-BD FEV_1_ from baseline

**Figure 5:**
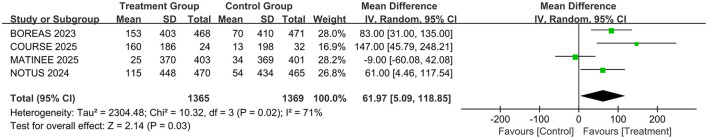
Forest plot of change in pre-BD FEV_1_ from baseline.

Data on the change from baseline in pre-BD FEV_1_ at 52 weeks were available from 4 RCTs (Figure 5). A random-effects meta-analysis demonstrated a statistically significant improvement in pre-BD FEV_1_ with type 2 mAbs compared with placebo (*k* = 4, *n* = 2,734; MD = 61.97 mL, 95% CI 5.09 to 118.85 mL; *P* = 0.03). Substantial heterogeneity was observed across studies (*I*^2^ = 71%, τ^2^ = 2304.48, *P* = 0.02), which may be attributable to differences in drug types and enrolment criteria.

#### 3.3.3 Quality of life: change in SGRQ score from baseline

**Figure 6:**
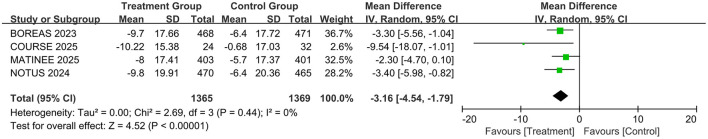
Forest plot of change in SGRQ score from baseline.

4 RCTs provided the change from baseline in SGRQ score at 52 weeks (Figure 6). A random-effects meta-analysis found that type 2 mAbs decreased the SGRQ score (*k* = 4, *n* = 2,734; MD = −3.16, 95% CI −4.54 to −1.79; *P* < 0.00001). No significant heterogeneity was observed (*I*^2^ = 0%, τ^2^ = 0, *P* = 0.44).

#### 3.3.4 Change in E-RS: COPD score from baseline

**Figure 7:**
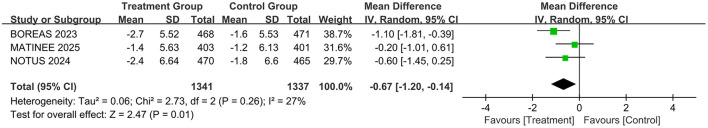
Forest plot of change in E-RS: COPD score from baseline.

The change from baseline in E-RS: COPD score at 52 weeks was assessed in 3 RCTs (Figure 7). Pooled analysis using a random-effects model revealed that type 2 mAbs lowered the E-RS: COPD score (*k* = 3, *n* = 2,678; MD = −0.67, 95% CI −1.20 to −0.14; *P* = 0.01). Heterogeneity was acceptable (*I*^2^ = 27%, τ^2^ = 0.06, *P* = 0.26). Despite the small number of included studies, the concordant and statistically significant results point to a modest symptom improvement with treatment.

#### 3.3.5 Safety endpoint: adverse events

**Figure 8:**
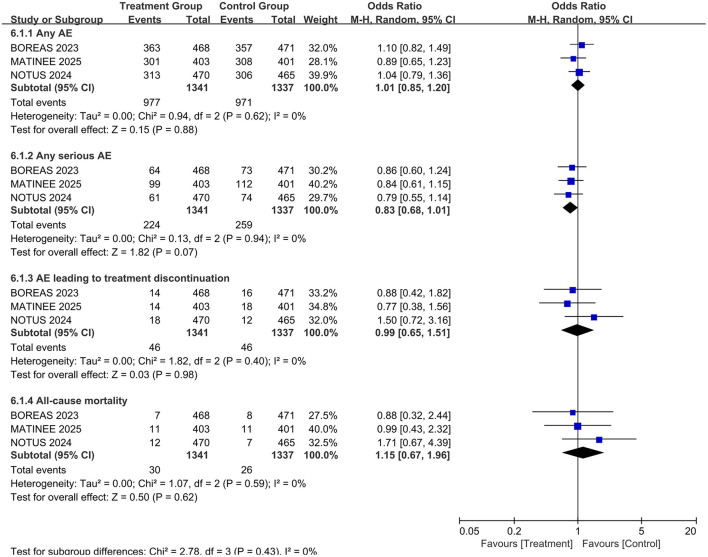
Forest plot of adverse events.

The safety analysis comprised 3 RCTs (Figure 8). The results for any adverse events (OR = 1.01, 95% CI 0.85 to 1.20, *P* = 0.88, *I*^2^ = 0%), serious adverse events (OR = 0.83, 95% CI 0.68 to 1.01, *P* = 0.07, *I*^2^ = 0%), discontinuation due to adverse events (OR = 0.99, 95% CI 0.65 to 1.51, *P* = 0.98, *I*^2^ = 0%) and all-cause mortality (OR = 1.15, 95% CI 0.67 to 1.96, *P* = 0.62, *I*^2^ = 0%) were consistent with no difference between the treatment group and the placebo group. The test for subgroup differences gave a *P* value of 0.43, indicating a similar overall trend across safety outcomes.

### 3.4 Sensitivity analysis and publication bias test

Several sensitivity analyses were performed to evaluate the robustness of the outcomes (Supplementary material). In the leave-one-out sensitivity analysis, the results of the annualized rate of moderate-to-severe exacerbations and SGRQ score remained stable across all exclusion scenarios, with no single study dominating the overall effect. For pre-BD FEV_1_, exclusion of the MATINEE trial reduced heterogeneity substantially (*I*^2^ from 71% to 5%), with the pooled estimate remaining statistically significant. However, when BOREAS, COURSE, or NOTUS was excluded, the confidence intervals crossed zero, indicating a loss of statistical significance. For E-RS: COPD score, the pooled estimate lost significance when BOREAS or NOTUS was excluded. These findings suggest that the FEV_1_ and E-RS: COPD results are more sensitive to the inclusion or exclusion of specific studies and should be interpreted with greater caution. Second, after exclusion of studies relying on historical eosinophil values, the re-analysis of the annualized moderate-to-severe exacerbation rate yielded findings consistent with the primary analysis. In addition, when a fixed effects model was applied to re-pool all outcomes, the results were highly comparable to those obtained with the random effects model. Regarding publication bias, because fewer than 10 studies were included, no funnel plot or Egger's regression test was performed, in accordance with the Cochrane Handbook. Hence, this study could not formally evaluate publication bias, but we tried to minimize such bias during the search stage by including conference abstracts and unpublished registered trials.

### 3.5 Certainty of evidence

The certainty of evidence for the outcomes was assessed using the GRADE approach, with results ranging from high to moderate. The annual rate of moderate-to-severe exacerbations, pre-BD FEV_1_, and E-RS: COPD were all rated as moderate certainty. The exacerbation rate was downgraded for indirectness due to the use of historical data, whereas pre-BD FEV_1_ and E-RS: COPD were downgraded for inconsistency, as sensitivity analyses was not robust. By contrast, the SGRQ achieved high certainty, with no factors warranting a downgrade. Detailed rating procedures and downgrading factors are presented in Table 3.

**Table 3:** GRADE evidence profile for outcomes of type 2 monoclonal antibodies vs. placebo in eosinophilic COPD patients.

Outcome	No. of research units (total participants^a^)	Risk of bias	Inconsistency	Indirectness	Imprecision	Publication bias	Overall certainty
Annual moderate-to-severe exacerbation rate	5 (3171)	Not serious	Not serious	Some concerns^b^	Not serious	Not assessed^c^	⊕⊕⊕○ Moderate
Change in pre-BD FEV_1_ (mL)	4 (2734)	Not serious	Serious^d^	Not serious	Not serious	Not assessed^c^	⊕⊕⊕○ Moderate
Change in SGRQ score	4 (2734)	Not serious	Not serious	Not serious	Not serious	Not assessed^c^	⊕⊕⊕⊕ High
E-RS: COPD	3 (2678)	Not serious	Serious^e^	Not serious	Not serious	Not assessed^c^	⊕⊕⊕○ Moderate

^a^The number of participants included in the meta-analysis, rather than the total number of participants in the trial.

^b^Indirectness: The results influenced by the use of historical BEC data (METREX/METREO) still rated indirectness as “some concerns” rather than “serious,” as sensitivity analysis confirmed robustness.

^c^Due to the inclusion of fewer than 10 studies, no statistical test for publication bias was conducted. However, a systematic search was conducted across multiple databases and conference abstracts.

^d^Due to significant heterogeneity (*I*^2^ = 71%), it was downgraded and this was confirmed through sensitivity analysis.

^e^The rating was downgraded due to the sensitivity analysis (where the *I*^2^ estimate was unstable when BOREAS or NOTUS were excluded).

## 4 Discussion

### 4.1 Principal findings

This meta-analysis pooled 6 RCTs involving 3,171 participants, precisely focused on patients with eosinophilic COPD, defined as BEC ≥300 cells/μL, and performed subgroup analyses to systematically evaluate the efficacy and safety of monoclonal antibodies targeting the type 2 inflammatory pathway, including dupilumab, mepolizumab, and tezepelumab. Overall, compared with placebo, type 2 mAbs reduced the risk of moderate-to-severe exacerbations in patients with COPD, and this benefit was consistently observed across different demographic characteristics and clinical subgroups. In addition, type 2 mAbs may show slight effects in improving lung function, enhancing quality of life, and alleviating daily symptoms, without increasing the occurrence of serious adverse events. It should be noted that the strict screening BEC ≥300 criterion was uniformly applied to all secondary outcomes, while the primary outcome used a broader definition because of the inclusion of METREX and METREO (whose included population included those who used historical BEC data); yet sensitivity analysis without historical-data trials confirmed its robustness and overall consistency. These findings confirm, from an evidence-based perspective, the rationale for targeting the eosinophilic phenotype as a precision treatment strategy in COPD, and provide a reliable basis for identifying suitable candidates for biologic therapy in clinical practice.

### 4.2 Regarding the population heterogeneity of “eosinophilic COPD”

Although a unified standard for defining eosinophilic COPD is currently lacking, the GOLD guidelines and most studies have identified BEC ≥300 cells/μL as the key threshold for recognizing type 2 inflammation and guiding biologic treatment (25, 26). On the basis of this threshold, several large scale RCTs have confirmed that type 2 mAbs substantially lower exacerbation rates and improve clinical outcomes in this population (13–15). However, in clinical practice, BEC exhibits temporal variability—studies have shown that approximately 74% of COPD patients have BEC levels that cross different clinical cutoffs (27). This variability implies that the inflammatory profile inferred from BEC status may differ according to the time of enrollment. Specifically, persistently elevated BEC could signal a stable and sustained type 2 inflammatory drive, whereas a history of high counts with current normal levels might reflect an intermittent or fluctuating state. From a mechanistic perspective, patients presenting with “current eosinophilia” would be predicted to gain more consistent and pronounced inflammatory blockade, while those with “historical eosinophilia” may show responses contingent on the activity of their underlying inflammatory status. In contrast to the mechanistic speculation, existing evidence suggests that this discrepancy may not compromise the overall direction of therapeutic benefit: a *post hoc* analysis of METREX/METREO revealed that patients with any documented BEC ≥300 cells/μL within the preceding year could derive benefit from treatment; similarly, a *post hoc* analysis of BOREAS/NOTUS indicated that treatment remained effective even when baseline BEC had declined (28, 29). Taken together, despite the differences in BEC stability between the two populations, both are likely to represent treatable traits associated with type 2 inflammation.

In this meta-analysis, both strict trials defined by a screening BEC ≥300 cells/μL (e.g., BOREAS, NOTUS, MATINEE) and more lenient ones based on a BEC ≥300 cells/μL within the year prior to enrollment (e.g., METREX, METREO) were included. Such heterogeneity in population definitions may bias the pooled effect estimates toward the average response of patients meeting the broader threshold, rather than capturing the specific effect of those with persistently elevated BEC. To gauge the effect of this definitional variation, we conducted a sensitivity analysis on the annualized rate of moderate-to-severe exacerbations, restricting the inclusion to the trials using a screening BEC ≥300 cells/μL. The estimates derived from this subset proved comparable to those of the primary analysis, indicating that heterogeneity attributable to differing BEC entry criteria did not materially affect the core findings. Accordingly, we judge pooling of the two populations as acceptable under the available evidence, but future studies ought to differentiate persistent from transient eosinophilia to yield more nuanced evidence for the optimized use of type 2 monoclonal antibodies.

### 4.3 Clinical implications and mechanism exploration

Acute exacerbations, particularly moderate-to-severe episodes, represent critical events in the course of COPD. They are closely associated with accelerated decline in lung function, deterioration in quality of life, and increased risk of hospitalization, thereby exerting a comprehensive negative impact on disease progression, patient health status, and mortality outcomes (30–32). Recognizing this, the Global Initiative for Chronic Obstructive Lung Disease (GOLD) has explicitly designated reducing the future risk of exacerbations as one of the two core goals in the management of stable COPD (26). Given that eosinophilic COPD exhibit a higher frequency of exacerbations, they represent a key target population for priority management in clinical practice. The present study shows that type 2 mAbs reduce the annualized exacerbation rate in eosinophilic COPD, a benefit that is of clear clinical importance by meaningfully decreasing both the healthcare burden and the risk of disease progression. Mechanistically, type 2 inflammation is critically involved in COPD exacerbations. Viral infection, air pollution, allergens and other triggers induce airway epithelial cells to release alarmins (such as TSLP and IL-33), which in turn activate T helper 2 cells (Th2 cells) and group 2 innate lymphoid cells (ILC2s) (33, 34). This leads to the substantial production of type 2 cytokines including IL-4, IL-5 and IL-13. These cytokines facilitate the mobilization, chemotaxis to the airways, and survival of eosinophils, and also stimulate mast cells and B cells to produce IgE, culminating in acute worsening of airway inflammation (35, 36). Type 2 mAbs block this cascade at different steps. Dupilumab inhibits both IL-4 and IL-13 signaling, thereby attenuating the Th2 immune response from an upstream level, whereas mepolizumab directly neutralizes IL-5 and reduces eosinophil production and activation. Tezepelumab acts at an even more upstream level and can suppress multiple inflammatory pathways, including both type 2 and non-type 2 pathways. Despite their distinct mechanisms of action, different type 2 mAbs effectively reduce the exacerbation frequency in eosinophilic COPD, indicating that the type 2 inflammatory pathway represents an important and actionable node in the disease progression of this patient subgroup (37).

It is worth noting that in the type 2 inflammatory cascade described above, IL-5, as a key effector cytokine generated by activated Th2 cells and ILC2s, serves as a critical link between upstream and downstream events. However, its functions extend beyond the promotion of eosinophilic inflammation (38). The functional IL-5 receptor is also expressed on various structural and immune cells, including airway epithelial cells, fibroblasts, mast cells, and plasma cells, suggesting a direct involvement of IL-5 in structural pathological changes—airway remodeling and epithelial barrier dysfunction—an effect independent of its regulation of eosinophil counts (39, 40). For instance, IL-5 upregulates inflammatory and fibrosis related cytokines in lung fibroblasts via signaling pathways like JAK2/STAT3, and promotes epithelial mesenchymal transition and extracellular matrix deposition, constituting the key pathological basis of airway remodeling (41, 42). Therefore, the clinical benefits of type 2 mAbs, especially those directly targeting IL-5 or its receptor, may derive in part from modulating these non-eosinophil mediated pathological processes, which offers a fresh perspective on their impact on lung function and airway structure. Of course, the current evidence stems largely from basic research and indirect findings in the asthma field, and its direct relevance to COPD remains to be further established.

FEV_1_ is the core parameter for assessing the severity of airflow limitation and lung function impairment in COPD. Another key observation from this study is the modest improvement in pre-BD FEV_1_ from baseline following type 2 mAbs treatment. Although the average improvement did not reach the minimal clinically important difference (MCID), considering the natural decline in lung function, even a moderate increase may delay lung function loss by 1–2 year (43, 44). Over a longer time horizon, this effect could translate into a reduction in mortality (45). Moreover, the improvement in lung function was highly heterogeneous, which may be related to differences in drug targets, baseline eosinophil levels, and follow-up durations. Notably, relative to mepolizumab, dupilumab demonstrated more prominent improvements in FEV_1_. An indirect comparison study found that dupilumab showed statistical significance in improving lung function, whereas mepolizumab did not (46). This difference may be attributed to their distinct mechanisms of action. Anti-IL-4Rα therapy reduces airway mucus hypersecretion and airway remodeling by blocking both IL-4 and IL-13, while anti-IL-5 therapy primarily focuses on eosinophil depletion, with relatively limited effects on airway structure (11, 47). Therefore, in patients with notable lung function impairment or symptoms of airway mucus hypersecretion, dupilumab may offer additional advantages.

Somewhat regrettably, in this study, type 2 mAbs led to some degree of improvement in patient-reported outcomes, yet the SGRQ score did not reach the generally accepted MCID threshold (48). This finding warrants a cautious, multi-faceted interpretation. On the one hand, patients with eosinophilic phenotypes have more frequent exacerbations and greater upper respiratory symptoms than those with non-eosinophilic COPD—that is, they have a higher baseline disease burden, which may compress the magnitude of improvement (49, 50). On the other hand, the MCID was primarily established based on traditional populations, and the group mean does not preclude the possibility that individual may derive substantial benefit.

Our subgroup analyses did not reveal any differential effects, a finding that may be related to the stringent inflammatory threshold used and the limited sample size within subgroups. Therefore, the possibility of efficacy differences in certain subgroups cannot be completely ruled out. Of note, in the subgroup with ≥3 exacerbations per year, moderate heterogeneity was observed, largely driven by the opposite direction of effect in the MATINEE trial; however, given that the treatment effects were directionally consistent across other exacerbation-frequency subgroups, this heterogeneity is most likely explained by the small sample size and accompanying random variation. Interestingly, from an observational trend perspective, the subgroup with BEC ≥500 cells/μL and the subgroup with ≥4 prior exacerbations per year showed lower rate ratios, suggesting a potential trend toward greater efficacy. Although the interaction tests did not reach statistical significance, these trends may still serve as important hypothesis-generating evidence for future research and warrant validation in larger prospective trials.

Across the 3 RCTs incorporated in this meta-analysis, the overall safety profile of type 2 monoclonal antibodies was comparable to that of placebo, with no additional safety signals detected. The incidence of serious adverse events was numerically lower in the treatment group, although this difference did not reach statistical significance. Overall, the current evidence supports a favorable short-term safety profile for type 2 monoclonal antibodies in eosinophilic COPD patients, but confirmation of their long-term safety will require studies with larger sample sizes and extended follow-up.

The results of the sensitivity analyses, particularly the leave-one-out analysis, require further discussion. The findings for pre-BD FEV_1_ and E-RS: COPD were less stable, warranting separate consideration. Heterogeneity for pre-BD FEV_1_ dropped markedly when MATINEE was excluded, implicating this trial as the main source of variability. This likely reflects mechanistic differences between drug classes. Mepolizumab (anti IL-5) primarily curbs eosinophil trafficking and survival, with benefit mainly through exacerbation reduction; dupilumab (anti IL-4Rα), by blocking IL-4/IL-13 (key drivers of airway remodeling), may yield more consistent FEV_1_ improvements (37). And the observed loss of statistical significance when omitting BOREAS, COURSE, or NOTUS is probably attributable to the increased relative weight of MATINEE rather than a true null effect. As for E-RS: COPD, only three trials contributed to the E-RS: COPD outcome, with BOREAS as the sole study showing a significant effect; NOTUS and MATINEE did not. The limited study count and heterogeneous point estimates render the pooled estimate unstable, and this finding should therefore be interpreted with caution. These observations do not alter the main conclusion on exacerbation reduction, but they imply larger studies to confirm the effects of type 2 mAbs on lung function and symptoms, and to better define the relative benefits of agents targeting distinct type 2 pathways.

### 4.4 Future research directions

Driven by the evolving understanding of heterogeneous phenotypes, distinct endotypes, and treatable traits, COPD has entered the era of precision medicine (51). Combining multiple biomarkers to identify responsive populations represents a future trend, and several studies have already begun to provide valuable clues in this direction. A *post hoc* analysis of the BOREAS study has made it clear that baseline BEC and FeNO concentration—both readily accessible biomarkers—are predictive of the response to dupilumab, with higher levels of each translating into progressively greater therapeutic gains (52). In parallel, a systematic review found that patients with eosinophilic phenotypes and respiratory comorbidities (e.g., chronic sinusitis) may derive more pronounced clinical improvements with type 2 mAbs, suggesting that comorbidity status should be incorporated into precision phenotyping (36). However, achieving true precision phenotyping remains challenging. Although biomarkers such as FeNO and BEC have shown predictive potential, large-scale, multicenter validation studies are still lacking to establish unified thresholds for clinical use (53, 54). Furthermore, the economic burden of testing for novel biomarkers is considerable — a factor that limits their accessibility to some extent (55). Therefore, future research should focus on integrating multi-omics data and multidimensional biomarkers, leveraging cutting-edge tools like artificial intelligence and machine learning, to develop more accurate and cost-effective predictive models for treatment response, ultimately advancing precision medicine in COPD (56, 57).

### 4.5 Strengths and limitations

Recent literature has explored the use of biologic agents in COPD (58–61). In contrast to those studies, the present study provides important updates and extensions to the existing evidence in following respects. In terms of evidence timeliness, our meta-analysis includes several recently published large-scale trials, including MATINEE, COURSE, and NOTUS, some of which were not captured in previous meta-analyses. Regarding population definition, we imposed a strict enrollment criterion of BEC ≥300 cells/μL, thereby concentrating on a core cohort driven by more explicit type 2 inflammation. This design circumvents the attenuation of effect estimates that could result from including patients with low eosinophil counts or mixed phenotypes, and sharpens the applicability of our conclusions to the target population. Furthermore, we imposed no restriction on the type of type 2 monoclonal antibodies, and instead included all relevant trials that met our eligibility criteria. In terms of analytical depth, we performed exploratory subgroup analyses across a range of clinically relevant variables, a strategy that has not been undertaken in prior research, with the goal of delivering richer evidence to inform individualized clinical decision making.

Several limitations deserve mention. First, only subgroup data were available from the COURSE, METREX and METREO trials, with missing baseline characteristics, leading to an incomplete description of the target population; the small sample size of the COURSE subgroup may also have reduced statistical power. Second, the patient population from the METREX and METREO study units included those based on historical BEC values ≥300 cells/μL, which diverges from the population definition of trials that directly measured BEC at screening. While temporal variability in BEC is a well-recognized feature of COPD and sensitivity analyses confirmed the robustness of the results, such a discrepancy may nonetheless introduce some degree of population heterogeneity. Third, the number of incorporated studies was relatively small, and neither funnel plots nor Egger's regression tests were conducted. In addition to the fact that publication bias cannot be fully excluded, the range of monoclonal antibodies covered was also limited—the analysis only involved type 2 monoclonal antibodies targeting the IL-4 Rα, IL-5, and TSLP pathways, with no biologics directed at other inflammatory pathways. This may restrict the generalizability of our findings. Fourth, most subgroup analyses included only 3 studies—a limited sample size that compromises statistical power and raises the risk of false-negative findings. Hence, larger-scale studies are needed for further validation.

## 5 Conclusions

This meta-analysis is the first to systematically evaluate the efficacy and safety of type 2 mAbs in eosinophilic COPD defined by an BEC of ≥300 cells/μL. The results showed that anti-type 2 biologic therapies reduced the rate of moderate-to-severe exacerbations and may be associated with modest improvements in lung function, quality of life, and daily symptoms, without increasing the risk of serious adverse events. In conclusion, for eosinophilic COPD patients who experience frequent exacerbations despite optimized inhaled therapy, type 2 mAbs represent an effective add-on treatment option. Nevertheless, future large scale, prospective, multicenter studies are still required to establish unified clinical thresholds and to advance precision phenotyping that integrates multiple biomarkers, thereby further optimizing individualized treatment strategies.

## Data Availability

The original contributions presented in the study are included in the article/Supplementary material, further inquiries can be directed to the corresponding author.
