# Supplementary material for: Anti-type 2 monoclonal antibody therapy for eosinophilic COPD beyond triple therapy: a systematic review and meta-analysis with strict eosinophil inclusion criteria
Source: Front Med (Lausanne). 2026 Jul 22;13:1895960. doi: 10.3389/fmed.2026.1895960 (PMC13437951; doi:10.3389/fmed.2026.1895960)
Supplement: Supplementary file 1 [file Table_1.docx]

**Supplementary Materials**

Urban Warming Reshapes the Temporal Windows for Safe Outdoor Activities: Evidence from Dual Indices of Heat_Risk_Hours and PAO (Supplementary Material)

This supplementary material provides the full set of results for all cities cited in the main text, metrics for seasonal structural shifts, city-specific external consistency validations, and robustness checks (addressing sensitivity to weighting schemes and trend estimation methods).

**Contents**

- Table S1. Core results for all 31 cities (2010–2024).
- Table S2. Season-shift (May–Sep) metrics for all 31 cities (2010–2024).
- Table S3. External consistency validation by city (September window).
- Table S4. Robustness: W_index weighting sensitivity (equal-weight vs PCA).
- Table S5. Robustness: trend estimation sensitivity (Theil–Sen vs OLS-HC3).

**Table S1. Core results for all 31 cities (2010–2024).**

| **W_rank_desc** | **City** | **Heat_Risk_Hours_mean** | **Heat_Risk_Hours_slope_per_year** | **PAO_Rate_mean** | **PAO_Rate_slope_per_year** | **W_index** | **Age65_share_mean_percent** | **Age65_share_slope_pp_per_year** | **Quadrant** |
| --- | --- | --- | --- | --- | --- | --- | --- | --- | --- |
| 1 | Wuhan | 1096 | 22.2 | 0.359 | -0.0048 | 2.904 | 10.71 | 0.368 | Double trap |
| 2 | Changsha | 1062 | 15.86 | 0.366 | -0.0071 | 2.61 | 10.49 | 0.208 | Climate-pressure |
| 3 | Haikou | 1123 | 17.4 | 0.135 | -0.006 | 2.495 | 8.21 | 0.162 | Climate-pressure |
| 4 | Chengdu | 733.8 | 13.33 | 0.542 | -0.0072 | 2.191 | 12.54 | 0.36 | Double trap |
| 5 | Chongqing | 1020 | 13.85 | 0.395 | -0.0066 | 2.084 | 15.47 | 0.536 | Double trap |
| 6 | Nanchang | 1078 | 15.08 | 0.33 | -0.0057 | 1.959 | 9.69 | 0.283 | Climate-pressure |
| 7 | Hefei | 878.6 | 16 | 0.432 | -0.005 | 1.889 | 11.15 | 0.28 | Climate-pressure |
| 8 | Hangzhou | 937.8 | 16.18 | 0.402 | -0.0048 | 1.824 | 10.87 | 0.264 | Climate-pressure |
| 9 | Shanghai | 548.4 | 11.73 | 0.522 | -0.0069 | 1.816 | 14.44 | 0.62 | Double trap |
| 10 | Zhengzhou | 836.9 | 12.25 | 0.504 | -0.0055 | 1.408 | 8.43 | 0.182 | Climate-pressure |
| 11 | Fuzhou | 937.1 | 12 | 0.32 | -0.0056 | 1.387 | 10.67 | 0.351 | Double trap |
| 12 | Shijiazhuang | 883.5 | 12.6 | 0.491 | -0.0033 | 0.685 | 11.44 | 0.473 | Double trap |
| 13 | Jinan | 787.8 | 12 | 0.503 | -0.0035 | 0.638 | 12.64 | 0.476 | Double trap |
| 14 | Nanning | 1162 | 6.86 | 0.225 | -0.0054 | 0.428 | 10.13 | 0.176 | Climate-pressure |
| 15 | Guangzhou | 1300 | 11.71 | 0.136 | -0.0025 | 0.229 | 7.48 | 0.115 | Climate-pressure |
| 16 | Tianjin | 713.5 | 10.5 | 0.514 | -0.003 | 0.194 | 13.74 | 0.336 | Double trap |
| 17 | Nanjing | 816.3 | 6.8 | 0.46 | -0.0045 | 0.071 | 12.34 | 0.452 | Double trap |
| 18 | Urumqi | 251.9 | 11.75 | 0.534 | -0.0011 | -0.249 | 8.12 | 0.336 | Aging-sensitive |
| 19 | Xi_an | 689.5 | 7.5 | 0.576 | -0.0026 | -0.486 | 10.17 | 0.244 | Lower-pressure |
| 20 | Taiyuan | 306.5 | 8.17 | 0.625 | -0.0019 | -0.605 | 9.83 | 0.27 | Lower-pressure |
| 21 | Guiyang | 83.5 | 4.75 | 0.766 | -0.0033 | -0.719 | 9.04 | 0.142 | Lower-pressure |
| 22 | Yinchuan | 370.5 | 9.6 | 0.582 | 0.0002 | -1.118 | 8.04 | 0.256 | Lower-pressure |
| 23 | Lanzhou | 306.9 | 8.75 | 0.612 | 0.0001 | -1.221 | 10.82 | 0.293 | Lower-pressure |
| 24 | Beijing | 790.1 | 5 | 0.502 | -0.0016 | -1.258 | 11.92 | 0.46 | Aging-sensitive |
| 25 | Shenyang | 321.1 | 6.17 | 0.59 | 0.0006 | -1.855 | 13.94 | 0.51 | Aging-sensitive |
| 26 | Kunming | 2.5 | 0.22 | 0.859 | -0.0004 | -2.525 | 9.85 | 0.212 | Lower-pressure |
| 27 | Harbin | 187.1 | 0.67 | 0.599 | 0.0004 | -2.753 | 12.68 | 0.656 | Aging-sensitive |
| 28 | Lhasa | 0 | 0 | 0.481 | 0.0001 | -2.761 | 5.29 | 0.085 | Lower-pressure |
| 29 | Changchun | 184 | 2.36 | 0.606 | 0.0014 | -2.791 | 12.33 | 0.607 | Aging-sensitive |
| 30 | Hohhot | 82.9 | 1 | 0.582 | 0.0016 | -3.127 | 10.66 | 0.432 | Aging-sensitive |
| 31 | Xining | 0 | 0 | 0.552 | 0.0018 | -3.346 | 9.34 | 0.26 | Lower-pressure |

*Note: Columns report city-level means and trend slopes for Heat_Risk_Hours and PAO_Rate, the composite worsening index (W_index), aging level (Age65_share_percent), and quadrant labels used in the main text.*

*Units: Heat_Risk_Hours in hours/year; slopes in h/year or per-year (PAO_Rate); Age65 slopes in percentage points/year.*

**Table S2. Season-shift (May–Sep) metrics for all 31 cities (2010–2024).**

| **Rank_Late33_slope_desc** | **Rank_Late35_slope_desc** | **City** | **Quadrant** | **LatePeak33_AugSep_share_2010_2024** | **Slope_LatePeak33_AugSep_perYear** | **LatePeak35_AugSep_share_2010_2024** | **Slope_LatePeak35_AugSep_perYear** | **SepPeak33_share_2010_2024** | **SepPeak35_share_2010_2024** | **Slope_PeakMonth33_perYear** | **Slope_PeakMonth35_perYear** |
| --- | --- | --- | --- | --- | --- | --- | --- | --- | --- | --- | --- |
| 1 | 2 | Wuhan | Double trap | 0.667 | 0.0607 | 0.733 | 0.0464 | 0.267 | 0.4 | 0.0929 | 0.0393 |
| 2 | 1 | Haikou | Climate-pressure | 0.4 | 0.0536 | 0.4 | 0.0536 | 0.267 | 0.267 | 0.15 | 0.1821 |
| 3 | 10 | Hangzhou | Climate-pressure | 0.667 | 0.05 | 0.667 | 0.0143 | 0.267 | 0.333 | 0.0464 | 0.0143 |
| 3 | 4 | Harbin | Aging-sensitive | 0.4 | 0.05 | 0.667 | 0.0429 | 0.333 | 0.667 | 0.1464 | 0.1321 |
| 5 | 10 | Changsha | Climate-pressure | 0.467 | 0.0393 | 0.533 | 0.0143 | 0.267 | 0.333 | 0.0571 | 0.025 |
| 6 | 23 | Nanchang | Climate-pressure | 0.533 | 0.0357 | 0.6 | -0.0107 | 0.267 | 0.333 | 0.025 | -0.0286 |
| 7 | 7 | Zhengzhou | Climate-pressure | 0.467 | 0.0286 | 0.4 | 0.0179 | 0.2 | 0.2 | 0.025 | 0 |
| 7 | 22 | Xi_an | Lower-pressure | 0.467 | 0.0286 | 0.333 | -0.0071 | 0.2 | 0.2 | 0.0179 | 0.0071 |
| 7 | 31 | Taiyuan | Lower-pressure | 0.333 | 0.0286 | 0.467 | -0.0357 | 0.2 | 0.467 | 0.0643 | -0.0607 |
| 7 | 2 | Hefei | Climate-pressure | 0.6 | 0.0286 | 0.667 | 0.0464 | 0 | 0.067 | 0.0286 | 0.0607 |
| 11 | 7 | Nanjing | Double trap | 0.6 | 0.025 | 0.667 | 0.0179 | 0.267 | 0.267 | 0.0214 | 0.0143 |
| 12 | 16 | Shenyang | Aging-sensitive | 0.533 | 0.0214 | 0.933 | 0.0071 | 0.267 | 0.8 | 0.0536 | 0.0214 |
| 13 | 27 | Yinchuan | Lower-pressure | 0.4 | 0.0143 | 0.4 | -0.025 | 0.267 | 0.333 | 0.0214 | -0.0143 |
| 14 | 10 | Shanghai | Double trap | 0.533 | 0.0107 | 0.6 | 0.0143 | 0.267 | 0.267 | 0.0071 | 0.0071 |
| 15 | 24 | Jinan | Double trap | 0.133 | 0.0071 | 0.267 | -0.0143 | 0 | 0.067 | 0 | -0.0393 |
| 16 | 7 | Chongqing | Double trap | 0.8 | 0.0036 | 0.733 | 0.0179 | 0.2 | 0.2 | 0.0071 | 0.0214 |
| 19 | 25 | Beijing | Aging-sensitive | 0.333 | -0 | 0.333 | -0.0179 | 0.267 | 0.333 | -0.0036 | -0.0643 |
| 17 | 18 | Lhasa | Lower-pressure | 1 | 0 | 1 | 0 | 1 | 1 | 0 | 0 |
| 17 | 18 | Kunming | Lower-pressure | 1 | 0 | 1 | 0 | 1 | 1 | 0 | 0 |
| 20 | 30 | Urumqi | Aging-sensitive | 0.6 | -0.0036 | 0.6 | -0.0321 | 0.267 | 0.333 | 0.025 | -0.0571 |
| 20 | 10 | Guangzhou | Climate-pressure | 0.8 | -0.0036 | 0.8 | 0.0143 | 0.467 | 0.533 | 0.0179 | 0.0393 |
| 22 | 6 | Xining | Lower-pressure | 0.8 | -0.0071 | 0.933 | 0.025 | 0.8 | 0.933 | -0.0143 | 0.05 |
| 22 | 16 | Lanzhou | Lower-pressure | 0.6 | -0.0071 | 0.867 | 0.0071 | 0.533 | 0.867 | -0.0321 | 0.0143 |
| 24 | 15 | Nanning | Climate-pressure | 0.667 | -0.0107 | 0.733 | 0.0107 | 0.467 | 0.533 | 0.0143 | 0.0571 |
| 25 | 18 | Shijiazhuang | Double trap | 0.067 | -0.0143 | 0 | 0 | 0 | 0 | -0.0643 | -0.0679 |
| 26 | 10 | Changchun | Aging-sensitive | 0.667 | -0.0214 | 0.867 | 0.0143 | 0.6 | 0.867 | -0.0464 | 0.0429 |
| 28 | 26 | Tianjin | Double trap | 0.133 | -0.025 | 0.133 | -0.0214 | 0 | 0 | -0.0429 | -0.0929 |
| 27 | 5 | Chengdu | Double trap | 0.8 | -0.025 | 0.667 | 0.025 | 0.4 | 0.4 | -0.0393 | 0.0321 |
| 29 | 18 | Guiyang | Lower-pressure | 0.867 | -0.0321 | 1 | 0 | 0.6 | 1 | -0.1429 | 0 |
| 30 | 29 | Hohhot | Aging-sensitive | 0.533 | -0.0464 | 0.733 | -0.0286 | 0.467 | 0.733 | -0.0821 | -0.0857 |
| 31 | 27 | Fuzhou | Double trap | 0.667 | -0.0536 | 0.467 | -0.025 | 0.267 | 0.267 | -0.0571 | -0.0286 |

*Note: Includes late-season peak shares (Aug–Sep), September-peak shares, and peak-month migration diagnostics for HotDays thresholds (≥33°C / ≥35°C).*

*These metrics support Figure 2 and the warm-season structure reallocation analysis in the Results.*

**Table S3. External consistency validation by city (September window).**

| **City** | **rho_Hours_vs_HotDays_33** | **p_Hours_vs_HotDays_33** | **rho_Hours_vs_HotDays_35** | **p_Hours_vs_HotDays_35** | **slope_UTCI_SeptRiskHours_perYear** | **slope_HotDays33_Sept_perYear** | **slope_HotDays35_Sept_perYear** | **Note** |
| --- | --- | --- | --- | --- | --- | --- | --- | --- |
| Beijing | 0 | 1 | — | — | 2.296 | 0.011 | 0 | Ties/constant series |
| Changchun | — | — | — | — | -0.093 | 0 | 0 | No variation / insufficient data |
| Changsha | 0.256 | 0.35682 | 0.221 | 0.42819 | 5.807 | 0.707 | 0.586 | — |
| Chengdu | 0.72 | 0.0024904 | 0.433 | 0.10691 | 4.771 | 0.368 | 0.175 | — |
| Chongqing | 0.647 | 0.0090839 | 0.764 | 0.00091717 | 7.589 | 0.975 | 0.657 | — |
| Fuzhou | 0.642 | 0.0098417 | 0.642 | 0.0098027 | 4.293 | 1.014 | 0.693 | — |
| Guangzhou | -0.128 | 0.65039 | 0.505 | 0.05485 | 3.589 | -0.179 | 0.239 | — |
| Guiyang | 0.095 | 0.73647 | — | — | 0.696 | 0.018 | 0 | — |
| Haikou | 0.098 | 0.72828 | -0.091 | 0.74719 | 4.361 | 0.3 | 0.007 | — |
| Hangzhou | 0.629 | 0.012021 | 0.636 | 0.010808 | 2.482 | 0.546 | 0.382 | — |
| Harbin | — | — | — | — | -0.179 | 0 | 0 | No variation / insufficient data |
| Hefei | 0.807 | 0.00028015 | 0.722 | 0.0023854 | 5.671 | 0.343 | 0.221 | — |
| Hohhot | — | — | — | — | 0 | 0 | 0 | No variation / insufficient data |
| Jinan | 0.158 | 0.57295 | — | — | 3.518 | 0.243 | 0 | — |
| Kunming | — | — | — | — | 0 | 0 | 0 | No variation / insufficient data |
| Lanzhou | — | — | — | — | 1.375 | 0 | 0 | No variation / insufficient data |
| Lhasa | — | — | — | — | 0 | 0 | 0 | No variation / insufficient data |
| Nanchang | 0.666 | 0.0066988 | 0.734 | 0.0018535 | 5.282 | 0.961 | 0.557 | — |
| Nanjing | 0.519 | 0.047467 | 0.699 | 0.0037017 | 4.554 | 0.368 | 0.104 | — |
| Nanning | 0.506 | 0.054552 | 0.524 | 0.044936 | 2.311 | 0.279 | 0.229 | — |
| Shanghai | 0.451 | 0.091801 | 0.595 | 0.019204 | 3.961 | 0.457 | 0.079 | — |
| Shenyang | — | — | — | — | 0.136 | 0 | 0 | No variation / insufficient data |
| Shijiazhuang | 0.582 | 0.022779 | 0.445 | 0.096474 | 3.768 | 0.161 | 0.036 | — |
| Taiyuan | -0.21 | 0.45342 | — | — | 0.536 | 0.029 | 0 | — |
| Tianjin | 0.535 | 0.039898 | 0.403 | 0.13659 | 2.829 | 0.086 | 0.007 | — |
| Urumqi | 0.749 | 0.0013198 | 0.375 | 0.16861 | 0.6 | 0.082 | 0.036 | — |
| Wuhan | 0.436 | 0.10456 | 0.51 | 0.051868 | 7.564 | 0.721 | 0.45 | — |
| Xi_an | 0.574 | 0.025168 | 0.433 | 0.10656 | 2.996 | 0.4 | 0.1 | — |
| Xining | — | — | — | — | 0 | 0 | 0 | No variation / insufficient data |
| Yinchuan | 0.612 | 0.015278 | — | — | 1.207 | 0.136 | 0 | — |
| Zhengzhou | 0.357 | 0.19106 | 0.124 | 0.65933 | 4.661 | 0.589 | 0.196 | — |

*Note: Spearman rank correlations (ρ) and p-values between September UTCI risk hours and station-based HotDays (≥33°C / ≥35°C), plus city-level trend slopes used for comparison.*

*Supports Figure 1B–C and the external-consistency statements in the main text.*

**Table S4. Robustness: W_index weighting sensitivity (equal-weight vs PCA).**

| **City** | **W_index_equal** | **W_index_PCA1** | **Rank_equal** | **Rank_PCA1** | **DeltaRank** |
| --- | --- | --- | --- | --- | --- |
| Wuhan | 2.9038 | 2.0533 | 1 | 1 | 0 |
| Changsha | 2.6104 | 1.8458 | 2 | 2 | 0 |
| Haikou | 2.4952 | 1.7644 | 3 | 3 | 0 |
| Chengdu | 2.1908 | 1.5491 | 4 | 4 | 0 |
| Chongqing | 2.0839 | 1.4735 | 5 | 5 | 0 |
| Nanchang | 1.9589 | 1.3851 | 6 | 6 | 0 |
| Hefei | 1.8893 | 1.3359 | 7 | 7 | 0 |
| Hangzhou | 1.8241 | 1.2898 | 8 | 8 | 0 |
| Shanghai | 1.816 | 1.2841 | 9 | 9 | 0 |
| Zhengzhou | 1.4083 | 0.99582 | 10 | 10 | 0 |
| Fuzhou | 1.3875 | 0.9811 | 11 | 11 | 0 |
| Shijiazhuang | 0.68458 | 0.48407 | 12 | 12 | 0 |
| Jinan | 0.63811 | 0.45121 | 13 | 13 | 0 |
| Nanning | 0.42813 | 0.30273 | 14 | 14 | 0 |
| Guangzhou | 0.22939 | 0.16221 | 15 | 15 | 0 |
| Tianjin | 0.19408 | 0.13723 | 16 | 16 | 0 |
| Nanjing | 0.071149 | 0.05031 | 17 | 17 | 0 |
| Urumqi | -0.24857 | -0.17576 | 18 | 18 | 0 |
| Xi_an | -0.48582 | -0.34353 | 19 | 19 | 0 |
| Taiyuan | -0.60457 | -0.4275 | 20 | 20 | 0 |
| Guiyang | -0.71934 | -0.50865 | 21 | 21 | 0 |
| Yinchuan | -1.1177 | -0.7903 | 22 | 22 | 0 |
| Lanzhou | -1.2209 | -0.86331 | 23 | 23 | 0 |
| Beijing | -1.2583 | -0.88972 | 24 | 24 | 0 |
| Shenyang | -1.8546 | -1.3114 | 25 | 25 | 0 |
| Kunming | -2.5254 | -1.7857 | 26 | 26 | 0 |
| Harbin | -2.7528 | -1.9465 | 27 | 27 | 0 |
| Lhasa | -2.7615 | -1.9527 | 28 | 28 | 0 |
| Changchun | -2.7913 | -1.9737 | 29 | 29 | 0 |
| Hohhot | -3.127 | -2.2111 | 30 | 30 | 0 |
| Xining | -3.3459 | -2.3659 | 31 | 31 | 0 |

*Note: Compares city rankings under the main-text equal-weight W_index and an alternative PCA-based weighting scheme.*

*ΔRank indicates sensitivity of tiering conclusions.*

**Table S5. Robustness: trend estimation sensitivity (Theil–Sen vs OLS-HC3).**

| **City** | **Sen_slope_Risk_h_per_yr** | **OLS_HC3_slope_Risk_h_per_yr** | **Sign_consistent_Risk** | **Sen_slope_PAO_per_yr** | **OLS_HC3_slope_PAO_per_yr** | **Sign_consistent_PAO** | **W_index_Sen_equal** | **W_index_OLS_equal** | **Rank_W_Sen** | **Rank_W_OLS** |
| --- | --- | --- | --- | --- | --- | --- | --- | --- | --- | --- |
| Wuhan | 22.2 | 22.725 | Yes | -0.0048277 | -0.005184 | Yes | 2.9038 | 2.8076 | 1 | 2 |
| Changsha | 15.857 | 15.089 | Yes | -0.0071301 | -0.0071565 | Yes | 2.6104 | 2.2164 | 2 | 4 |
| Haikou | 17.4 | 21.404 | Yes | -0.006047 | -0.00683 | Yes | 2.4952 | 3.145 | 3 | 1 |
| Chengdu | 13.333 | 13.954 | Yes | -0.0071973 | -0.0074891 | Yes | 2.1908 | 2.1417 | 4 | 5 |
| Chongqing | 13.846 | 12.271 | Yes | -0.0066449 | -0.0068365 | Yes | 2.0839 | 1.6451 | 5 | 8 |
| Nanchang | 15.083 | 16.054 | Yes | -0.0056849 | -0.0056606 | Yes | 1.9589 | 1.8709 | 6 | 7 |
| Hefei | 16 | 17.743 | Yes | -0.0050381 | -0.0065894 | Yes | 1.8893 | 2.4618 | 7 | 3 |
| Hangzhou | 16.182 | 14.521 | Yes | -0.0047658 | -0.0055351 | Yes | 1.8241 | 1.5766 | 8 | 9 |
| Shanghai | 11.727 | 12.904 | Yes | -0.0069379 | -0.0078296 | Yes | 1.816 | 2.0838 | 9 | 6 |
| Zhengzhou | 12.25 | 13.193 | Yes | -0.0055374 | -0.0046967 | Yes | 1.4083 | 1.0755 | 10 | 12 |
| Fuzhou | 12 | 12.686 | Yes | -0.0056022 | -0.0055692 | Yes | 1.3875 | 1.2862 | 11 | 11 |
| Shijiazhuang | 12.6 | 10.621 | Yes | -0.0033361 | -0.0033964 | Yes | 0.68458 | 0.21434 | 12 | 16 |
| Jinan | 12 | 11.464 | Yes | -0.0035014 | -0.0037154 | Yes | 0.63811 | 0.46049 | 13 | 14 |
| Nanning | 6.8571 | 8.5071 | Yes | -0.0054466 | -0.0063969 | Yes | 0.42813 | 0.87779 | 14 | 13 |
| Guangzhou | 11.714 | 10.846 | Yes | -0.0024964 | -0.0030851 | Yes | 0.22939 | 0.14646 | 15 | 17 |
| Tianjin | 10.5 | 10.643 | Yes | -0.0029956 | -0.0036453 | Yes | 0.19408 | 0.30178 | 16 | 15 |
| Nanjing | 6.8 | 13.914 | Yes | -0.004474 | -0.0052511 | Yes | 0.071149 | 1.381 | 17 | 10 |
| Urumqi | 11.75 | 11.218 | Yes | -0.0011388 | -0.0010514 | Yes | -0.24857 | -0.47784 | 18 | 18 |
| Xi_an | 7.5 | 7.4821 | Yes | -0.0025677 | -0.002272 | Yes | -0.48582 | -0.68093 | 19 | 20 |
| Taiyuan | 8.1667 | 8.9607 | Yes | -0.0019063 | -0.0016262 | Yes | -0.60457 | -0.65538 | 20 | 19 |
| Guiyang | 4.75 | 5.0107 | Yes | -0.003268 | -0.0019199 | Yes | -0.71934 | -1.2061 | 21 | 22 |
| Yinchuan | 9.6 | 10.121 | Yes | 0.00023829 | -6.9055e-05 | No | -1.1177 | -0.98925 | 22 | 21 |
| Lanzhou | 8.75 | 7.9107 | Yes | 0.00010893 | -0.00031999 | No | -1.2209 | -1.2683 | 23 | 23 |
| Beijing | 5 | 3.7036 | Yes | -0.001634 | -0.0018412 | Yes | -1.2583 | -1.4476 | 24 | 24 |
| Shenyang | 6.1667 | 5.4357 | Yes | 0.00061275 | -9.5316e-05 | No | -1.8546 | -1.7511 | 25 | 25 |
| Kunming | 0.22222 | 0.38214 | Yes | -0.00043573 | -0.00059038 | Yes | -2.5254 | -2.4155 | 26 | 26 |
| Harbin | 0.66667 | 0.43214 | Yes | 0.00042088 | 0.00031124 | Yes | -2.7528 | -2.7111 | 27 | 28 |
| Lhasa | 0 | 0 | Yes | 0.00011671 | 0.00036466 | Yes | -2.7615 | -2.8002 | 28 | 29 |
| Changchun | 2.3571 | 2.7857 | Yes | 0.0013617 | 0.0009658 | Yes | -2.7913 | -2.5446 | 29 | 27 |
| Hohhot | 1 | -0.52143 | No | 0.001634 | 0.0016077 | Yes | -3.127 | -3.3049 | 30 | 30 |
| Xining | 0 | 0 | Yes | 0.001755 | 0.0022623 | Yes | -3.3459 | -3.4397 | 31 | 31 |

*Note: Compares city-level trend slopes estimated via Theil–Sen (main text) against OLS with heteroskedasticity-robust standard errors (HC3).*

*Includes sign-consistency indicators where available.*
