# Supplementary material for: Anti-type 2 monoclonal antibody therapy for eosinophilic COPD beyond triple therapy: a systematic review and meta-analysis with strict eosinophil inclusion criteria
Source: Front Med (Lausanne). 2026 Jul 22;13:1895960. doi: 10.3389/fmed.2026.1895960 (PMC13437951; doi:10.3389/fmed.2026.1895960)
Supplement: Supplementary file 2 [file Table_2.docx]

***Supplementary Materials***

**Anti–Type 2 Monoclonal Antibody Therapy for Eosinophilic COPD Beyond Triple Therapy: A Systematic Review and Meta-Analysis with Strict Eosinophil Inclusion Criteria**

**Supplementary Material 1 - Systematic search details**

**TABLE 1 Pubmed**

| Sequence | Search | Hits |
| --- | --- | --- |
| #1 | Pulmonary Disease, Chronic Obstructive[MeSH Terms] | 74770 |
| #2 | ((((((((((Pulmonary Disease, Chronic Obstructive[Title/Abstract]) OR (Chronic Obstructive Pulmonary Diseases[Title/Abstract])) OR (COPD[Title/Abstract])) OR (Chronic Obstructive Lung Disease[Title/Abstract])) OR (Chronic Obstructive Pulmonary Disease[Title/Abstract])) OR (COAD[Title/Abstract])) OR (Chronic Obstructive Airway Disease[Title/Abstract])) OR (Airflow Obstruction, Chronic[Title/Abstract])) OR (Airflow Obstructions, Chronic[Title/Abstract])) OR (Chronic Airflow Obstructions[Title/Abstract])) OR (Chronic Airflow Obstruction[Title/Abstract]) | 100702 |
| #3 | (#1) OR (#2) | 122748 |
| #4 | Antibodies, Monoclonal[MeSH Terms] | 306371 |
| #5 | (((((((((((((((((((((((((((((((Antibodies, Monoclonal[Title/Abstract]) OR (Monoclonal Antibody[Title/Abstract])) OR (Antibody, Monoclonal[Title/Abstract])) OR (Monoclonal Antibodies[Title/Abstract])) OR (mepolizumab[Title/Abstract])) OR (bosatria[Title/Abstract])) OR (nucala[Title/Abstract])) OR (sb-240563[Title/Abstract])) OR (sb240563[Title/Abstract])) OR (reslizumab[Title/Abstract])) OR (cinqaero[Title/Abstract])) OR (cinqair[Title/Abstract])) OR (sch-55700[Title/Abstract])) OR (sch55700[Title/Abstract])) OR (benralizumab[Title/Abstract])) OR (fasenra[Title/Abstract])) OR (medi-563[Title/Abstract])) OR (medi563[Title/Abstract])) OR (dupilumab[Title/Abstract])) OR (GSK679586[Title/Abstract])) OR (tezepelumab[Title/Abstract])) OR (TSLP[Title/Abstract])) OR (tozorakimab[Title/Abstract])) OR (itepekimab[Title/Abstract])) OR (astegolimab[Title/Abstract])) OR (IL-4[Title/Abstract])) OR (IL4[Title/Abstract])) OR (IL-13[Title/Abstract])) OR (IL-5[Title/Abstract])) OR (IL5[Title/Abstract])) OR (IL-33[Title/Abstract])) OR (IL33[Title/Abstract]) | 304737 |
| #6 | (#4) OR (#5) | 480339 |
| #7 | ((((((randomized controlled trial[Publication Type]) OR (randomized controlled trial[Title/Abstract])) OR (RCT[Title/Abstract])) OR (clinical trial[Title/Abstract])) OR (controlled trial[Title/Abstract])) OR (randomized[Title/Abstract])) OR (placebo[Title/Abstract]) | 1333796 |
| #8 | ((#3) AND (#6)) AND (#7) | 146 |

**TABLE 2 Embase**

| Sequence | Search | Hits |
| --- | --- | --- |
| #1 | 'chronic obstructive lung disease'/exp | 224655 |
| #2 | 'chronic obstructive lung disease':ab,ti OR 'chronic airflow obstruction':ab,ti OR 'chronic airway obstruction':ab,ti OR 'chronic obstructive bronchopulmonary disease':ab,ti OR 'chronic obstructive lung disorder':ab,ti OR 'chronic obstructive pulmonary disease':ab,ti OR 'chronic obstructive pulmonary disorder':ab,ti OR 'chronic obstructive respiratory disease':ab,ti OR 'chronic pulmonary obstructive disease':ab,ti OR 'chronic pulmonary obstructive disorder':ab,ti OR copd:ab,ti OR 'lung chronic obstructive disease':ab,ti OR 'lung disease, chronic obstructive':ab,ti OR 'obstructive chronic lung disease':ab,ti OR 'obstructive chronic pulmonary disease':ab,ti OR 'obstructive lung disease, chronic':ab,ti OR 'pulmonary disease, chronic obstructive':ab,ti OR 'pulmonary disorder, chronic obstructive':ab,ti | 176079 |
| #3 | #1 OR #2 | 252939 |
| #4 | 'monoclonal antibody'/exp | 978302 |
| #5 | 'monoclonal antibody':ab,ti OR 'antibodies, monoclonal':ab,ti OR 'antibodies, monoclonal, humanized':ab,ti OR 'antibodies, monoclonal, murine derived':ab,ti OR 'antibodies, monoclonal, murine-derived':ab,ti OR 'antibody, monoclonal':ab,ti OR 'clonal antibody':ab,ti OR 'hybridoma antibody':ab,ti OR 'monoclonal antibodies':ab,ti OR mepolizumab:ab,ti OR bosatria:ab,ti OR nucala:ab,ti OR 'sb-240563':ab,ti OR sb240563:ab,ti OR reslizumab:ab,ti OR cinqaero:ab,ti OR cinqair:ab,ti OR 'sch-55700':ab,ti OR sch55700:ab,ti OR benralizumab:ab,ti OR fasenra:ab,ti OR 'medi-563':ab,ti OR medi563:ab,ti OR dupilumab:ab,ti OR gsk679586:ab,ti OR tezepelumab:ab,ti OR tslp:ab,ti OR tozorakimab:ab,ti OR itepekimab:ab,ti OR astegolimab:ab,ti OR 'il 4':ab,ti OR il4:ab,ti OR 'il 13':ab,ti OR il13:ab,ti OR 'il 5':ab,ti OR il5:ab,ti OR 'il 33':ab,ti OR il33:ab,ti | 413292 |
| #6 | #4 OR #5 | 1157803 |
| #7 | 'randomized controlled trial':it OR 'randomized controlled trial':ab,ti OR rct:ab,ti OR 'clinical trial':ab,ti OR 'controlled trial':ab,ti OR randomized:ab,ti OR placebo:ab,ti | 1872557 |
| #8 | #3 AND #6 AND #7 | 753 |

**TABLE 3 Web of science**

| Sequence | Search | Hits |
| --- | --- | --- |
| #1 | TS=(Pulmonary Disease, Chronic Obstructive OR chronic obstructive lung disease OR chronic airflow obstruction OR chronic airway obstruction OR chronic obstructive bronchopulmonary disease OR chronic obstructive lung disorder OR chronic obstructive pulmonary disease OR chronic obstructive pulmonary disorder OR chronic obstructive respiratory disease OR chronic pulmonary obstructive disease OR chronic pulmonary obstructive disorder OR copd OR lung chronic obstructive disease OR lung disease, chronic obstructive OR obstructive chronic lung disease OR obstructive chronic pulmonary disease OR obstructive lung disease, chronic OR pulmonary disease, chronic obstructive OR pulmonary disorder, chronic obstructive) | 192292 |
| #2 | TS=(monoclonal antibody OR antibodies, monoclonal OR antibodies, monoclonal, humanized OR antibodies, monoclonal, murine derived OR antibodies, monoclonal, murine-derived OR antibody, monoclonal OR clonal antibody OR hybridoma antibody OR monoclonal antibodies OR mepolizumab OR bosatria OR nucala OR sb-240563 OR sb240563 OR reslizumab OR cinqaero OR cinqair OR sch-55700 OR sch55700 OR benralizumab OR fasenra OR medi-563 OR medi563 OR dupilumab OR gsk679586 OR tezepelumab OR TSLP OR tozorakimab OR itepekimab OR astegolimab OR IL-4 OR IL4 OR IL-13 OR IL13 OR IL-5 OR IL5 OR IL-33 OR IL33) | 728668 |
| #3 | TS=(randomized controlled trial OR RCT OR clinical trial OR controlled trial OR randomized OR placebo) | 3271782 |
| #4 | #3 AND #2 AND #1 | 502 |

**TABLE 4 Cochrane**

| Sequence | Search | Hits |
| --- | --- | --- |
| #1 | MeSH descriptor: [Pulmonary Disease, Chronic Obstructive] explode all trees | 8303 |
| #2 | (Pulmonary Disease, Chronic Obstructive):ti,ab,kw OR (Chronic Obstructive Pulmonary Disease):ti,ab,kw OR (Chronic Obstructive Airway Disease):ti,ab,kw OR (COPD):ti,ab,kw OR (COAD):ti,ab,kw OR (Chronic Obstructive Pulmonary Diseases):ti,ab,kw OR (Chronic Obstructive Lung Disease):ti,ab,kw OR (Chronic Airflow Obstruction):ti,ab,kw OR (Airflow Obstructions, Chronic):ti,ab,kw OR (Chronic Airflow Obstructions):ti,ab,kw OR (Airflow Obstruction, Chronic):ti,ab,kw | 26642 |
| #3 | #1 OR #2 | 26986 |
| #4 | MeSH descriptor: [Antibodies, Monoclonal] explode all trees | 23235 |
| #5 | (Antibodies, Monoclonal):ti,ab,kw OR (Antibody, Monoclonal):ti,ab,kw OR (Monoclonal Antibodies):ti,ab,kw OR (Monoclonal Antibody) OR (mepolizumab):ti,ab,kw OR (bosatria):ti,ab,kw OR (nucala):ti,ab,kw OR (sb-240563):ti,ab,kw OR (sb240563):ti,ab,kw OR (reslizumab):ti,ab,kw OR (cinqaero):ti,ab,kw OR (cinqair):ti,ab,kw OR (sch-55700):ti,ab,kw OR (sch55700):ti,ab,kw OR (benralizumab):ti,ab,kw OR (fasenra):ti,ab,kw OR (medi-563):ti,ab,kw OR (medi563):ti,ab,kw OR (dupilumab):ti,ab,kw OR (gsk679586):ti,ab,kw OR (tezepelumab):ti,ab,kw OR (TSLP):ti,ab,kw OR (tozorakimab):ti,ab,kw OR (itepekimab):ti,ab,kw OR (astegolimab):ti,ab,kw OR (IL-4):ti,ab,kw OR (IL4):ti,ab,kw OR (IL-13):ti,ab,kw OR (IL13):ti,ab,kw OR (IL-5):ti,ab,kw OR (IL5):ti,ab,kw OR (IL-33):ti,ab,kw OR (IL33):ti,ab,kw | 31076 |
| #6 | #4 OR #5 | 39797 |
| #7 | MeSH descriptor: [Randomized Controlled Trial] explode all trees | 34 |
| #8 | (randomized controlled trial):ti,ab,kw OR (RCT):ti,ab,kw OR (clinical trial):ti,ab,kw OR (controlled trial):ti,ab,kw OR (randomized):ti,ab,kw OR (placebo):ti,ab,kw | 1509701 |
| #9 | #7 OR #8 | 1509701 |
| #10 | #3 AND #6 AND #9 in Trials | 355 |

**TABLE 5 CNKI**

| Sequence | Search | Hits |
| --- | --- | --- |
| #1 | (SU%=肺疾病,慢性阻塞性) OR TKA=(肺疾病,慢性阻塞性 + 慢性阻塞肺疾病 + COAD + 慢性道阻塞性疾病+ 慢性阻塞性肺疾病 + 慢性气流阻塞 + 慢性气道阻塞 + 气道阻塞,慢性 + COPD) | 238608 |
| #2 | (SU%=抗体,单克隆) OR TKA=(mepolizumab + reslizumab + benralizumab + astegolimab + itepekimab + dupilumab + tezepelumab + tozorakimab) OR TKA=(抗体,单克隆 + 单克隆抗体 + 美泊利单抗 + 瑞利珠单抗 + 贝那利珠单抗 + 阿斯特戈利单抗 + 伊特佩基单抗 + 度普利尤单抗 + 替西普利尤单抗) | 182968 |
| #3 | (SU%=随机对照试验) OR TKA=(随机对照试验 + 随机对照实验 + RCT + 安慰剂) | 650093 |
| #4 | #1 + #2 + #3 | 91 |

**TABLE 6 Wangfang**

| Sequence | Search | Hits |
| --- | --- | --- |
| #1 | 主题:(肺疾病,慢性阻塞性) OR 题名或关键词:(慢性阻塞肺疾病 OR COAD OR 慢性气道阻塞性疾病OR 慢性阻塞性肺疾病 OR 慢性气流阻塞 OR 慢性气道阻塞 OR 气道阻塞,慢性 OR COPD) | 160052 |
| #2 | 主题:(抗体,单克隆) OR 题名或关键词:(抗体,单克隆 OR 单克隆抗体 OR 美泊利单抗 OR 瑞利珠单抗 OR 贝那利珠单抗 OR 阿斯特戈利单抗 OR 伊特佩基单抗 OR 度普利尤单抗 OR 替西普利尤单抗 OR mepolizumab OR reslizumab OR benralizumab OR astegolimab OR itepekimab OR dupilumab OR tezepelumab OR tozorakimab) | 398717 |
| #3 | 主题:(随机对照试验) OR 题名或关键词:(随机对照试验 OR 随机对照实验 OR RCT OR 安慰剂) | 298860 |
| #4 | #1 AND #2 AND #3 | 1 |

**TABLE 7 VIP**

| Sequence | Search | Hits |
| --- | --- | --- |
| #1 | M=肺疾病,慢性阻塞性 OR 慢性阻塞肺疾病 OR COAD OR 慢性气道阻塞性疾病OR 慢性阻塞性肺疾病 OR 慢性气流阻塞 OR 慢性气道阻塞 OR 气道阻塞,慢性 OR COPD | 76787 |
| #2 | M=抗体,单克隆 OR 单克隆抗体 OR 美泊利单抗 OR 瑞利珠单抗 OR 贝那利珠单抗 OR 阿斯特戈利单抗 OR 伊特佩基单抗 OR 度普利尤单抗 OR 替西普利尤单抗 OR mepolizumab OR reslizumab OR benralizumab OR astegolimab OR itepekimab OR dupilumab OR tezepelumab OR tozorakimab | 20098 |
| #3 | R=随机对照试验 OR 随机对照实验 OR RCT OR 安慰剂 | 68402 |
| #4 | #1 AND #2 AND #3 | 1 |

**TABLE 8 CBM**

| Sequence | Search | Hits |
| --- | --- | --- |
| #1 | "肺疾病, 慢性阻塞性"[不加权:扩展] OR "慢性阻塞肺疾病"[常用字段:智能] OR "COAD"[常用字段:智能] OR "慢性气道阻塞性疾病"[常用字段:智能] OR "慢性阻塞性肺疾病"[常用字段:智能] OR "慢性气流阻塞"[常用字段:智能] OR "慢性气道阻塞"[常用字段:智能] OR "气道阻塞,"[常用字段:智能] AND "慢性"[常用字段:智能] OR "COPD"[常用字段:智能] | 213742 |
| #2 | "抗体, 单克隆"[不加权:扩展] OR "单克隆抗体"[常用字段:智能] OR "美泊利单抗"[常用字段:智能] OR "瑞利珠单抗"[常用字段:智能] OR "贝那利珠单抗"[常用字段:智能] OR "阿斯特戈利单抗"[常用字段:智能] OR "伊特佩基单抗"[常用字段:智能] OR "度普利尤单抗"[常用字段:智能] OR "替西普利尤单抗"[常用字段:智能] OR "mepolizumab"[常用字段:智能] OR "reslizumab"[常用字段:智能] OR "benralizumab"[常用字段:智能] OR "astegolimab"[常用字段:智能] OR "itepekimab"[常用字段:智能] OR "dupilumab"[常用字段:智能] OR "tezepelumab"[常用字段:智能] OR "tozorakimab"[常用字段:智能] | 392625 |
| #3 | "随机对照试验"[不加权:扩展] OR "随机对照试验"[常用字段:智能] OR "随机对照实验"[常用字段:智能] OR "RCT"[常用字段:智能] OR "安慰剂"[常用字段:智能] | 568946 |
| #4 | #1 AND #2 AND #3 | 3 |

**Supplementary Material 2 - Reasons for exclusion of full-text articles**

**TABLE 1**

| **ID** | **Study** | Trial Number Identifier | **Exclusion Reason** |
| --- | --- | --- | --- |
| 1 | Brightling, C. E. (2014) | NCT01227278 | Inappropriate participant |
| 2 | Criner, G. J. (2017) | NCT02138916  NCT02155660 | Inappropriate participant |
| 3 | Rabe, K. F. (2021) | NCT03546907 | Inappropriate participant |
| 4 | Yousuf, A. J. (2022) | NCT03615040 | Inappropriate participant |
| 5 | Singh, D. (2025) | NCT04631016 | Inappropriate participant |
| 6 | Ramakrishnan, S. (2025) | NCT04098718 | Inappropriate participant |
| 7 | Dasgupta, A. (2017) | NCT01463644 | Inappropriate outcome |
| 8 | Reid, F. (2024) | NCT03096795 | Inappropriate outcome |
| 9 | Flynn, C. A. (2025) | NCT04075331 | Inappropriate outcome |
| 10 | / | NCT04882124 | Trial registrations without results |
| 11 | / | NCT04053634 | Trial registrations without results |
| 12 | / | NCT06897748 | Trial registrations without results |
| 13 | / | NCT02546700 | Trial registrations without results |
| 14 | / | NCT06040086 | Trial registrations without results |
| 15 | / | NCT05158387 | Trial registrations without results |
| 16 | / | NCT05166889 | Trial registrations without results |
| 17 | / | NCT05595642 | Trial registrations without results |
| 18 | / | NCT05037929 | Trial registrations without results |
| 19 | / | NCT04701983  NCT04751487 | Trial registrations without results |
| 20 | / | CTRI/2022/12/047957 | Trial registrations without results |
| 21 | / | EUCTR2013-004297-98-NL | Trial registrations without results |
| 22 | / | ChiCTR2500104536 | Trial registrations without results |
| 23 | / | EUCTR2013‐004298‐28‐SE | Trial registrations without results |
| 24 | / | NL‐OMON44215 | Trial registrations without results |
| 25 | / | PER‐013‐14 | Trial registrations without results |
| 26 | / | NCT07059091 | Ongoing trials |
| 27 | / | NCT05878769 | Ongoing trials |
| 28 | / | NCT06883305  NCT06878261 | Ongoing trials |
| 29 | / | NCT06547333 | Ongoing trials |
| 30 | / | NCT07053423 | Ongoing trials |
| 31 | / | NCT06961214  NCT06959095 | Ongoing trials |
| 32 | / | NCT05507242 | Ongoing trials |
| 33 | / | EUCTR2018-003924-35-GB | Ongoing trials |
| 34 | / | CTRI/2025/05/086621 | Ongoing trials |
| 35 | / | CTRI/2024/06/069046 | Ongoing trials |

**Supplementary Material 3 - Sensitivity analysis**

**TABLE 1 Leave-one-out sensitivity analysis for annualized rate of moderate or severe exacerbations**

| Omitted Study | Rate Ratio | 95% CI | P value | *I²* (%) | τ² |
| --- | --- | --- | --- | --- | --- |
| None (overall) | 0.73 | 0.66 to 0.80 | <0.00001 | 0 | 0 |
| BOREAS 2023 | 0.74 | 0.66 to 0.83 | <0.00001 | 0 | 0 |
| COURSE 2025 | 0.73 | 0.67 to 0.81 | <0.00001 | 0 | 0 |
| MATINEE 2025 | 0.71 | 0.63 to 0.79 | <0.00001 | 0 | 0 |
| METREO + METREX 2017 | 0.72 | 0.64 to 0.80 | <0.00001 | 0 | 0 |
| NOTUS 2024 | 0.75 | 0.67 to 0.84 | <0.00001 | 0 | 0 |

**TABLE 2 Leave-one-out sensitivity analysis for change in pre-BD FEV_1_**

| Omitted Study | MD (ml) | 95% CI | P value | I² (%) | τ² |
| --- | --- | --- | --- | --- | --- |
| None (overall) | 61.97 | 5.09 to 118.85 | 0.03 | 71 | 2304.48 |
| BOREAS 2023 | 56.55 | -21.30 to 134.39 | 0.15 | 76 | 3488.45 |
| COURSE 2025 | 44.54 | -11.40 to 100.48 | 0.12 | 70 | 1706.94 |
| MATINEE 2025 | 82.60 | 45.45 to 119.76 | <0.0001 | 5 | 64.45 |
| NOTUS 2024 | 65.83 | -17.54 to 149.19 | 0.12 | 80 | 4216.27 |

**TABLE 3 Leave-one-out sensitivity analysis for change in SGRQ score**

| Omitted Study | MD | 95% CI | P value | I² (%) | τ² |
| --- | --- | --- | --- | --- | --- |
| None (overall) | -3.16 | -4.54 to -1.79 | <0.00001 | 0 | 0 |
| BOREAS 2023 | -3.23 | -5.39 to -1.08 | 0.003 | 25 | 0.96 |
| COURSE 2025 | -3.00 | -4.38 to -1.61 | <0.0001 | 0 | 0 |
| MATINEE 2025 | -3.58 | -5.25 to -1.91 | <0.0001 | 0 | 0 |
| NOTUS 2024 | -3.18 | -5.20 to -1.16 | 0.002 | 24 | 0.83 |

**TABLE 4 Leave-one-out sensitivity analysis for change in E-RS: COPD score**

| Omitted Study | MD | 95% CI | P value | I² (%) | τ² |
| --- | --- | --- | --- | --- | --- |
| None (overall) | -0.67 | -1.20 to -0.14 | 0.01 | 27 | 0.06 |
| BOREAS 2023 | -0.39 | -0.98 to 0.20 | 0.19 | 0 | 0 |
| MATINEE 2025 | -0.90 | -1.44 to -0.35 | 0.001 | 0 | 0 |
| NOTUS 2024 | -0.67 | -1.55 to 0.21 | 0.13 | 63 | 0.25 |

**TABLE 5 Comparison of calculation results between FEM and REM**

| Outcomes | RR/MD IV, Random, 95%CI | RR/MD IV, Fixed, 95%CI |
| --- | --- | --- |
| Annualized rate of moderate or severe exacerbations | 0.73 (0.66 to 0.80) | 0.73 (0.66 to 0.80) |
| Change in pre-FEV_1_ | 61.97 (5.09 to 118.85) | 52.15 (22.83 to 81.47) |
| Change in SGRQ total score | -3.16 (-4.54 to -1.79) | -3.16 (-4.54 to -1.79) |
| Change in E-RS: COPD total score | -0.67 (-1.20 to -0.14) | -0.68 (-1.13 to -0.23) |

**TABLE 6 Comparison of calculation results between all studies and those with screening‑only BEC ≥300 cells/μL***

| Outcomes | All studies | Those with screening‑only BEC ≥300 cells/μL |
| --- | --- | --- |
| Annualized rate of moderate or severe exacerbations | RR = 0.73 (0.66 to 0.80), P < 0.00001, I² = 0% | RR = 0.72 (0.64 to 0.80), P < 0.00001, I² = 0% |

***** Given that only the annualized rate of moderate or severe exacerbations involved historical data, sensitivity analysis was restricted to this outcome alone.
